# Herpes in the Heart: A Case of Widely Disseminated Intrauterine Herpes Simplex Virus Infection Involving Neonatal Myocardium in a 23-Week Gestationally Aged Neonate

**DOI:** 10.1155/2020/1305915

**Published:** 2020-08-28

**Authors:** Hirsch K. Srivastava, Lindsey T. Ellis, Douglas C. Miller, Deiter J. Duff

**Affiliations:** ^1^Department of Pathology & Anatomical Sciences, University of Missouri School of Medicine, Columbia, MO, USA; ^2^Department of Obstetrics, Gynecology & Women's Health, University of Missouri School of Medicine, Columbia, MO, USA

## Abstract

We report a rare case of disseminated herpes simplex virus (HSV) infection in an extremely preterm neonate. Herpes Simplex Virus-2 (HSV-2) is the leading cause of genital ulcer disease in adults and is the most common cause of neonatal herpes, a rare infection associated with long-term neurologic impairment and high mortality. HSV-2 can be transmitted perinatally via direct mucosal or skin contact. Most neonates are infected intrapartum. However, intrauterine transmission does occur, though rarely. The pattern of dissemination described in our patient differs from previous case reports. Most reports indicate that intrauterine HSV infections have a typical triad of cutaneous manifestations, ophthalmologic findings, and neurologic involvement. However, we report the first case of intrauterine disseminated HSV infection in the heart.

## 1. Background

Herpes simplex virus (HSV) infection of newborns can be acquired *in utero*, intrapartum, or postnatally. Herpes simplex virus 2 (HSV-2) is the leading cause of genital ulcer disease in adults and is the most common cause of neonatal herpes, a rare infection associated with long-term neurologic impairment and high mortality [1]. HSV-2 can be transmitted perinatally via direct mucosal or skin contact. Therefore, most affected neonates are infected intrapartum [1].

Primary and recurrent maternal infection during pregnancy can lead to intrauterine viral transmission resulting in a rare congenital disease, accounting for just 4-5% of all infections caused by HSV in neonates [2]. Data suggest that the incidence of neonatal HSV is 1 case in 3200 deliveries [3]. The incidence of intrauterine HSV infection is much less common, with an approximate frequency of 1 in 100,000 deliveries [3].

Though the incidence of intrauterine herpes is quite low, manifestations such as congenital brain malformation or severe neurocognitive impairment can lead to devastating consequences including fetal or neonatal death. The case fatality rate is 60%, and at least half of the survivors have either significant neurologic and/or ocular sequelae. Most reports indicate that intrauterine HSV infections have a typical triad of cutaneous manifestations, ophthalmologic findings, and neurologic involvement [4].

We illustrate a severe and unusual case of intrauterine HSV infection in a second-trimester neonate, discuss the limitations of the current definitions of intrauterine/intrapartum HSV, and confirm recent findings of the devastating effects of disseminated neonatal HSV infection. To our knowledge there have been few, if any, reports of disseminated HSV infection at this gestational age and no reports of disseminated HSV involving the heart.

## 2. Case

A 26-year-old G4P3 woman with a history of methylene tetrahydrofolate reductase (MTHFR) mutation and previous delivery of a microcephalic infant with a chromosomal abnormality presented for fetal ultrasound at a gestational age of 23 weeks, 2 days. An ultrasound indicated absent/reversed end diastolic flow, severe intrauterine growth restriction, and a fetal bradycardia. She was admitted to the Labor and Delivery Unit and given betamethasone for fetal lung maturity.

At the time of admission, she denied tobacco, drug, and alcohol use, and her urine drug screen was negative. Her prenatal serological tests were negative for HSV-1, HSV-2, HIV, chlamydia, syphilis, hepatitis B, and *Neisseria gonorrhoeae*. She was immune to Rubella. Toxoplasma IgG antibodies and CMV IgM and IgG antibodies were positive.

A cesarean delivery was performed shortly after admission due to non-reassuring fetal status. After delivery of the female infant, it was noted that the infant's skin was sloughing and had numerous large vesicles. At 1 minute of life, she was limp and blue with a heart rate of 20 beats per minute. Intubation attempts were made at 2 and 6 minutes of life, with successful intubation performed at 6.5 minutes of life. She was given surfactant at 8–10 minutes of life. Her heart rate remained <60 beats per minute, and she remained cyanotic despite administration of 100% FiO_2_. At 12.5 minutes of life, the family requested life-saving measures be discontinued. The infant's time of death was just under two hours after delivery. As per the family's request, an autopsy was performed, and the placenta was evaluated for any abnormalities.

**Figure 1:**
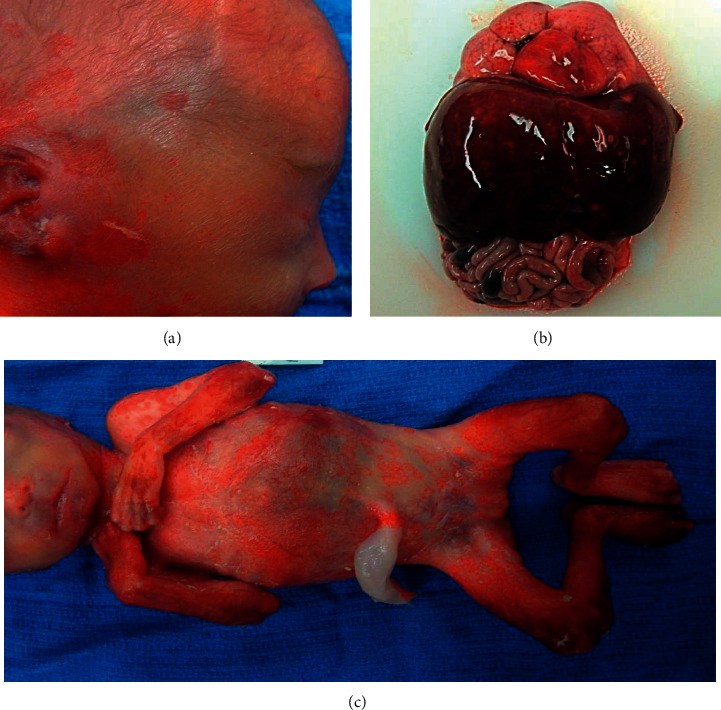
(a) Skin ulcerations of face and ear. (b) Numerous tan-yellow lesions of the liver. (c) Annular lesions of the abdominal skin and denuded extremities.

External examination revealed a well-developed, preterm female neonate with extensive skin sloughing and erythematous annular skin lesions with large portions of the body denuded (Figures 1(a) and 1(c)). There were no other significant external findings except those consistent with preterm birth.

Internal examination revealed approximately 2.0 mL of straw-colored fluid in the right thoracic cavity and 0.5 mL of similar fluid in the left thoracic cavity. The heart, spleen, lymph nodes, respiratory system, pancreas, genitourinary system, gastrointestinal system, and endocrine system were unremarkable. The liver capsule was smooth and glistening but had innumerable tan-yellow, round lesions up to 0.2 cm in diameter on the capsular surface; similar lesions were disseminated in the parenchyma (Figure 1(b)). The brain was friable and lacked gyral/sulcal development but was otherwise unremarkable.

**Figure 2:**
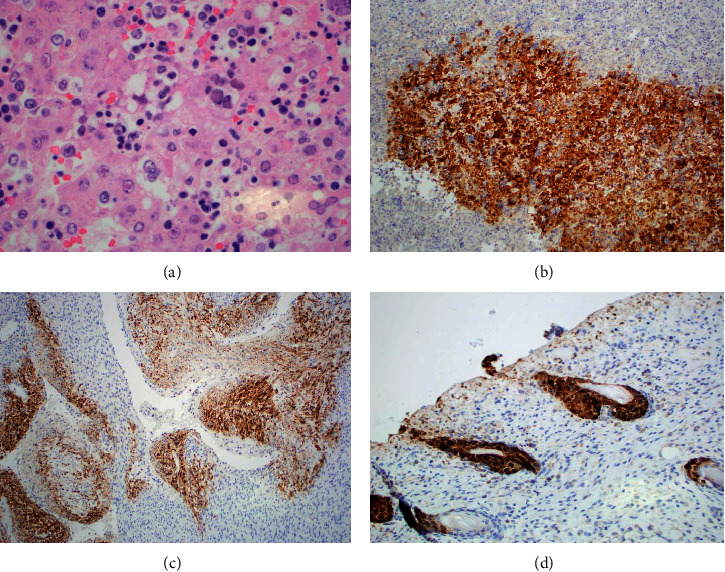
Liver: (a) Cowdry bodies and cytologic changes consistent with herpes simplex virus infection (H&E, ×600); (b) focus of infection (herpes simplex virus immunostain, ×100). Cardiac papillary muscle: (c) foci of infection (herpes simplex virus immunostain, ×100). Skin: (d) infection of hair follicle (herpes simplex virus immunostain, ×100).

Microscopic evaluation revealed dramatic histological changes affecting the liver, heart, and skin. The liver lesions revealed numerous areas of necrosis with large, multinucleate cells with margination and molding of their nuclei and Cowdry bodies that were immunopositive for HSV (using an HSV1/HSV2 antibody cocktail) (Figure 2(a)). The skin and myocardium had similar cytopathologic changes and necrosis (Figures 2(c) and 2(d)). The thymus, spleen, stomach, adrenal glands, and lungs also contained foci with weak cytoplasmic immunopositivity for HSV but with minimal cytopathologic changes. There was necrosis of the muscularis propria of the large bowel.

Microscopic examination of the brain did not reveal evidence of HSV infection. The cerebral sections lacked myelin (as seen with Luxol fast blue stains) consistent with an estimated gestational age of less than 22 weeks. The placental sections revealed immature placental architecture, subchorionic laminar decidual necrosis, and chronic villitis.

The infant's mother had no history of HSV infection and was asymptomatic at the time of delivery. A sample of the mother's blood from the time of delivery was negative by a PCR technique for HSV-1 and HSV-2.

## 3. Discussion

The neonate presented here died as a result of disseminated herpes simplex virus (HSV) infection acquired *in utero*. At the time of cesarean delivery, her skin was covered with large vesicular lesions and partially denuded. Histologic sections from skin and various other organs showed cytopathologic changes consistent with HSV infection. Additionally, sections of the heart, thymus, liver, spleen, and skin had cells that were immunopositive for HSV. While no definitive evidence of HSV infection was seen within the placenta, placental sections with subchorionic laminar decidual necrosis and chronic villitis can be associated with HSV infection [5].

While PCR is the most powerful method for detecting HSV, a negative result does not preclude infection [6]. In this case, the neonate's mother had a negative PCR result. However, the characteristic findings throughout the neonatal tissues with immunohistochemical staining for HSV-1/HSV-2 seen in this case suggest that the mother's negative PCR result is a false negative.

This case of widely disseminated intrauterine HSV infection illustrates the devastating effects of HSV while contrasting the clinical features seen in neonatal infections. For decades, neonatal HSV has been associated with the triad of cutaneous findings, ocular findings, and CNS involvement [4].

More recent studies, however, have found that this triad exists in only 30–39% of cases [2]. Previous studies showed that disseminated disease occurred in one half to two-thirds of all neonates with HSV exposure, however, that figure has been reduced to about 25% since the advent and utilization of antiviral therapy [4].

The frequencies of common manifestations of intrauterine HSV infections were reviewed by Marquez et al. in 2011. Their data suggest that 95% of cases have cutaneous manifestations [3]. The case described here did have skin manifestations, but we found no ocular or CNS involvement. Marquez et al. also showed that dissemination of HSV to the viscera occurs in approximately 35% of cases [3]. The disseminated infection presented here notably includes the liver, which is the most common site of such dissemination, as well as the adrenal glands and lungs, infection of which has also been reported. However, the most unique site of dissemination in this case was the heart. We have been unable to find previous cases of dissemination to heart tissue as confirmed here by immunochemistry.

## 4. Conclusions

Intrauterine herpes simplex virus (HSV) infection remains a rare but devastating disease. Only 5% of vertical HSV transmissions are intrauterine, representing approximately 1 in 300,000 deliveries. This dramatic case illustrates an extremely rare case of intrauterine HSV in that the infection involved the myocardial tissue. To the best of our knowledge, there have been few or no reported cases of disseminated HSV infection at this age, and no other cases of confirmed dissemination to the heart.

## Data Availability

No data were used to support this study.
